# An Experiment on Prediction Markets in Science

**DOI:** 10.1371/journal.pone.0008500

**Published:** 2009-12-30

**Authors:** Johan Almenberg, Ken Kittlitz, Thomas Pfeiffer

**Affiliations:** 1 Stockholm School of Economics, Stockholm, Sweden; 2 Consensus Point, Nashville, Tennessee, United States of America; 3 Program for Evolutionary Dynamics, Harvard University, Cambridge, Massachusetts, United States of America; George Mason University, United States of America

## Abstract

Prediction markets are powerful forecasting tools. They have the potential to aggregate private information, to generate and disseminate a consensus among the market participants, and to provide incentives for information acquisition. These market functionalities can be very valuable for scientific research. Here, we report an experiment that examines the compatibility of prediction markets with the current practice of scientific publication. We investigated three settings. In the first setting, different pieces of information were disclosed to the public during the experiment. In the second setting, participants received private information. In the third setting, each piece of information was private at first, but was subsequently disclosed to the public. An automated, subsidizing market maker provided additional incentives for trading and mitigated liquidity problems. We find that the third setting combines the advantages of the first and second settings. Market performance was as good as in the setting with public information, and better than in the setting with private information. In contrast to the first setting, participants could benefit from information advantages. Thus the publication of information does not detract from the functionality of prediction markets. We conclude that for integrating prediction markets into the practice of scientific research it is of advantage to use subsidizing market makers, and to keep markets aligned with current publication practice.

## Introduction

A prediction market is a marketplace for contracts whose payoffs depend on the outcome of a future event. In a well-functioning market, contract prices can be interpreted as forecasts about the outcome of the event, derived from the beliefs of all market participants. Contract types can be designed to elicit various aspects of the probability distribution associated with an event [1]. One popular contract type, for example, pays $1 if a specific outcome is realized, and $0 otherwise. The price of such a contract can be interpreted as the predicted probability of that outcome occurring. In practice, prediction markets facilitate trading by generating standardized contract rules, and are typically organized so that the market forecast is salient and easily interpreted.

To the extent that market prices can be interpreted as collective forecast, prediction markets disseminate, or broadcast, information. Although the mapping from individual beliefs to market prices is potentially complicated because individuals may differ in their risk aversion and in the availability of funds for betting [2], [3], in practice prediction markets have been found to generate good predictions for events ranging from product sales and horse races to presidential elections [4], [5]. By making collective forecasts available to a broader public, the dissemination property of prediction markets has the potential to generate social utility.

Prediction markets can also facilitate more complex information processing tasks. If different market participants have different, complementary pieces of private information, prediction markets have the potential to aggregate this information. Aggregation of dispersed information means that the market prediction is close to the forecast of a hypothetical trader in possession of all the information. A market that aggregates all available information is said to display strong efficiency [6], [7]. The information aggregation property is illustrated by an example from Plott (1988) [8]: Suppose an event has three mutually exclusive outcomes, X, Y, and Z. The payoff of a contract depends on which outcome is realized. Half of the traders in the market are informed that the outcome will not be X, and the other half is informed that the outcome will not be Y. A market that is able to aggregate this information will forecast that the outcome will be Z. This prediction differs from simply averaging the traders' initial beliefs. Information aggregation requires that traders learn from the market. Laboratory experiments suggest markets can accomplish information aggregation tasks reasonably well, although the details of the process are not fully understood [8], [9].

Because making reliable predictions is a key objective in science, prediction markets offer potential benefits to scientific research [10], [11]. Dissemination and aggregation properties of markets might be valuable because knowledge in scientific research is often highly decentralized. When settling a research problem, this may lead to diverging opinions within the research community. Prediction markets yield a consensus estimate on a scientific question that is communicated to market participants as well as to outside parties such as funding agencies and policy makers. The consensus disseminated by a functioning market has the potential to be more precise than a consensus obtained from traditional methods such as performing a meta-analysis of more or less biased data from the literature, or sampling expert opinion. A reliable consensus is important in order to generate agreement on what the most important open questions are and thus helps to allocate resources in an optimal way [12].

Prediction markets can also be used to fund research. Scientists that invest in a well-designed research project will gain an *information advantage*, i.e., their knowledge will be more accurate than the current consensus. In a prediction market, this information advantage can be used to re-capture the investment. Incentives can be amplified by subsidizing market makers that turn the zero-sum payoff structure of typical markets into a nonzero-sum game. Institutions that fund scientific research might therefore use prediction markets as an alternative to established ways of allocating resources. Instead of distributing funds based on past performance of researchers, they can use subsidized markets to efficiently allocate funds to researchers according to their actual contribution to a research problem. By rewarding an instantaneous, honest and unbiased disclosure of research findings, prediction markets may therefore help to overcome problems arising from publication-based incentives in the current practice of research, such as publication bias [13], [14] and delayed information disclosure. However, given the pivotal role of publishing in scientific research it is in our view crucial for potential applications of prediction markets to ensure compatibility with publishing.

As a first step toward the integration of prediction markets into scientific research practices, we designed an experimental prediction market around a stylized process of scientific hypothesis testing and publishing. Hypothesis testing was presented to the participants within the context of a simple biochemical testing problem that has been used in previous experiments on decision-making in science [12]. We presented six mutually exclusive hypotheses about a hypothetical biochemical pathway, and six different tests giving binary results that helped to identify the correct hypothesis. For each test, a positive result supported two of the hypotheses. Tests gave error-prone results, i.e. occasionally failed to provide a positive result for the true hypothesis (false negative) or provided positive evidence for a false hypothesis (false positive). The error rates were common knowledge. Further details on the hypotheses and tests are given in the Methods section.

Participants traded contracts representing the six mutually exclusive hypotheses. In each experimental market, 6 or 7 participants traded on a web-based prediction market interface comparable to commercially available market platforms. A subsidizing market-maker based on a logarithmic scoring rule [15], [16] was used to ensure liquidity despite the small number of traders, and to provide additional incentives for trading. Traders received a performance-independent fee of $15 in addition to the earnings from the market. Earnings from the market ranged from$1 to $40 and had a mean of about $14. Further details on participants, automated market maker and subsidies are given in the Methods section.

**Figure 1:**
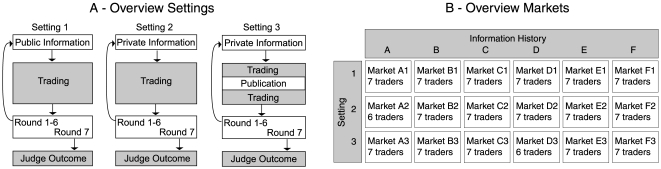
Experimental Setup. (A) In each market, participants traded claims representing six mutually exclusive hypotheses in seven consecutive trading rounds. At the outset of a trading round, information about the hypotheses in the form an error-prone test result, was disclosed either to all participants (Setting 1), or to an individual participant drawn at random (Settings 2 and 3). In Setting 3, the information was first private but then disclosed to the public. Each trading round took approximately three minutes. Trading was suspended for about two minutes between the trading rounds when novel information was distributed, and in the middle of each trading round in Setting 3, when private information was made public. After seven such trading rounds, the outcome was judged and the traders' accounts were settled. An entire session, including instructions and training on the market interface, took approximately 1 hr. **(B)** We used six different, randomly generated information histories (A–F), each of which differed in the tests and results given to the participants. The information history used in the market shown in Fig 2 is, for example, given by: OZ false, VO false, VZ false, OV true, ZV false, ZO true, VO true. Other information histories are given in the Methods. Each of the six information histories was used once in each of the three settings, giving in total 18 markets. Each trader participated in only one market, which means that the markets were not affected by differential learning effects.

The participants could trade during 7 trading rounds. Before each round, new information on the hypotheses was distributed in form of a test result. We investigated three different settings that differed in the way how information was distributed (see Fig 1A). In Setting 1, information was always public. All participants received the same test result at the same time, and could buy and sell contracts in the following trading round. In Setting 2, information was always private. In each round, only one of the participants received a test result. This participant was chosen randomly before the experiment, and was determined independently for each round. Some of the participants therefore received more than one test result while others received no information over the course of an experiment. As in Setting 1, each disclosure was followed by a trading round. In Setting 3, each piece of information was initially private, but eventually became public. One participant, drawn at random, received a test result before the trading round, just like in Setting 2. But halfway through the trading round the market was briefly suspended while the result was published, i.e. disclosed to all participants. We used six different “information histories”, each of which differed in the tests and results that were distributed (see Methods). Each of these six information histories was used once in each setting, resulting in 18 different markets (Fig 1B). Each participant could participate in only one market.

## Results

**Figure 2:**
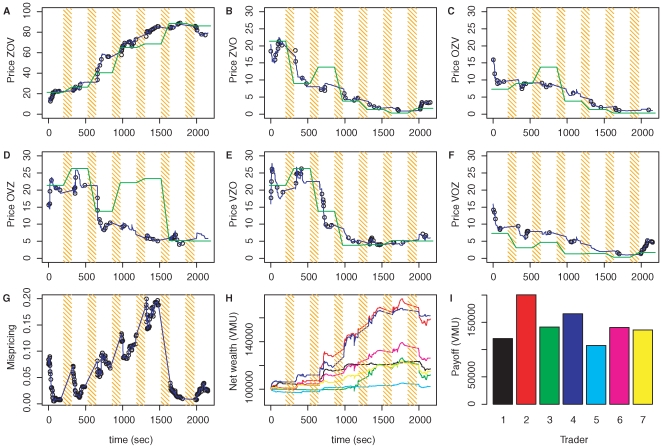
Example Market. **(A)–(F)** Prices of the six contracts over the course of one experiment session in Setting 1. Black dots indicate the trades in a share. Black lines show the theoretical market maker prices for infinitely small trades. The green line shows the correct pricing. Between the round (orange bars), trading is suspended, and new information is disclosed to the participants. **(G)** Mispricing. To quantify mispricing, we calculate the Kullback-Leibler divergence between the probability distribution implied by the market prices and the correct Bayesian posterior. Mispricing is typically generated when new information is released, and is subsequently reduced by the traders (see for example trading round 1 and 2). As it becomes more and more likely that the first hypothesis (ZOV) is the correct one, the traders in this session start overshooting in their estimate for the probability of this first hypothesis. This generates increasing mispricing in rounds 3–5. In round 6, the disclosed information reduces mispricing. This means that in this round, reality (i.e. correct prices) catches up with the market forecast. Prices at the end of the last round give a good estimate of the correct probabilities. **(H)** Net wealth of the seven traders in the market. The participants start with a cash position of 100,000, equivalent to USD 10. Over the course of the experiment they invest into the shares and thereby change the pricing of the shares and their own net wealth. **(I)** Final payoffs. After the last round, the hypotheses are judged: for each share of the correct hypotheses, participants receive a payoff of 100. This payoff is added to their cash position and determines the payoff. In this session all participants end up with a net gain. This illustrates the non-zero sum nature of trading due to the subsidizing market maker.

One of the markets in Setting 1 is shown in Fig 2. We observe that participants trade at high frequency. On average, each participant traded more than 35 times over the course of the experiment. Other markets showed similarly high trading activities. Participants traded even in the absence of private information, as observed in previous experimental asset markets [17], [18]. A lower trading volume than the one observed in the markets would have been sufficient to generate a correct pricing. Although all information was public and liquidity was high, we observe differences between the market prices and probabilities of the hypotheses as obtained by Bayesian updating (Fig 2G). This mispricing likely reflects the participants' limitations in information processing and Bayesian updating. However, in general contract prices approximately followed rational pricing, and the final pricing was sufficiently precise for all participants to extract a net profit from the market maker.

**Figure 3:**
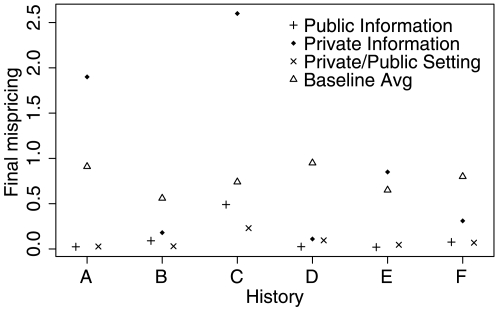
Mispricing after the final trading round. Market efficiency, measured as the mispricing at the end of the final trading round, was considerably higher in the setting with public information (Setting 1) than in the setting with private information (Setting 2). The setting with private information that was later made public (Setting 3) performed similar to the market with public information only (Setting 1). In the setting with private information, prices in three markets gave better forecasts than average trader belief. In the three remaining markets, average trader belief would have given better forecasts. Mispricing is shown as Kullback-Leibler distance. Predictions in well-functioning markets typically fall within a 5–10% margin around the correct probabilities. In the non-functioning markets (A2, C2, E2) prices differed from the correct probabilities by as much as 50–80%. In other words, predictions in these markets were far away from the correct probabilities and gave, for example, probabilities of 10% instead of 90% for the correct hypothesis.

To quantify mispricing and compare the overall performance of the market in the different settings, the Kullback-Leibler divergence 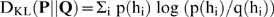 is calculated between a vector 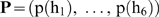 containing the probabilities of the hypotheses as obtained from Bayesian updating, and a vector 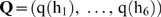 containing the actual prices of the contracts in the market using market maker prices [19]. This measure of mispricing is proportional to the profit of a rational trader who knows the correct probabilities and trades against the market maker used in our experiments [15], [16], [20]. Final mispricing, i.e. mispricing at the end of the last trading round, is shown for all 18 markets in Fig 3. To compare mispricing between different settings we use two-tailed paired t-tests on log-transformed final mispricing. While mispricing in Setting 3 was similar to mispricing in Setting 1 (t = −0.15, *p*≈0.89), mispricing in Setting 2 was higher than in the two other settings (Setting 1 vs. Setting 2: t = −3.7, p≈0.01; Setting 3 vs. Setting 2 t = −3.8, *p*≈0.01). We log-transform because a Shapiro-Francia test rejects normality of the mispricing but not of the log of mispricing (*p*-value 0.22).

Because in Setting 1 and 3, all information becomes public at some point, the market disseminates public information, but is not required to aggregate private information. Thus in both settings final mispricing should only depend on the participants' ability to estimate the correct probabilities. One would therefore not expect systematic differences in the performance in both setting, which is indeed what we observe. Setting 2, in contrast, requires the aggregation of private information. This task is more complex then the simple dissemination of information. In line with this, markets in Setting 2 show higher final mispricing for all of the six information histories.

In order to estimate whether the markets in Setting 2 aggregated private information, we compare the observed market forecasts against the average belief of a set of (hypothetical) rational individuals that all receive the same information as the market participants, do the correct Bayesian updating, but do not interact in a marketplace. In three of the markets, such an average belief would give a better forecast than market prices (see Fig 3). Thus, these markets clearly fail to aggregate information correctly. In the three remaining markets, prices produced forecasts that were considerably better than average belief. For these markets, performance falls into the range of the performance in the settings with public information. This indicates that the markets are in principle capable of aggregating information, but sometimes fail to do so.

In Setting 2 and 3, participants have private information, at least for some time. For these settings we can investigate whether participants can gain a profit from having an information advantage. This is important if prediction markets are to provide incentives to invest into research to gain an information advantage. We assess the relation between the profit and the information received by a participant based on two quantities, namely the *support* that a test provides for the true hypothesis, and its *informativity*. Both quantities account for the fact that the value of a piece of information depends on the information that precedes it. To give an extreme example, if the true hypothesis is already known with certainty, then additional information is of no value.

The support that a test result gives for the correct hypothesis can be quantified as 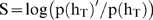, where p(h_T_) is the prior and p(h_T_)′ is the posterior probability of the correct hypothesis. Findings that maximize the posterior probability of the correct hypothesis are the most valuable ones. The support 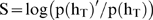 is proportional to the reward for increasing the probability of the correct hypothesis from p(h_T_) to p(h_T_)′ as provided by the market maker used in our experiment. However, calculating S requires knowing the correct hypotheses. A trader in our experiments can only assess the expected value of S, given by the Kullback-Leibler distance between the prior and posterior probability distribution **P** and **P′**, 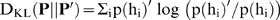. This informativity measure is equivalent to the mispricing measure used above and here quantifies mispricing from the viewpoint of a trader who received novel information, and determines the expected profit that can be gathered based on the information advantage.

Analysis of the payoffs reveals that the correlation between support S and net profit is stronger than the correlation between informativity and net profit: While the correlation between payoff (in virtual money units) and informativity was positive but not statistically significant (Setting 2: OLS, coefficient on informativity: 79,895, *p*-value≈0.2; Setting 3: OLS, coefficient on informativity: 49090, *p*-value≈0.6), we find a statistically significant positive correlation between payoff and support in both Setting 2 (OLS, coefficient on support: 47,039, *p*-value≈0.04) and Setting 3 (OLS, coefficient on support: 101,040, *p*-value < 0.01). This result means that in both settings, participants gained a profit from having an information advantage, particularly when the information was correct.

In a post-experiment questionnaire, subjects self-reported their familiarity with Bayes' rule and in a separate question were asked to make a Bayesian inference. We generated a binary variable that took on the value 1 if subjects reported being familiar with Bayes' rule and/or made the correct Bayesian inference. We observe a positive relation between this measure and net profit. The effect is statistically significant in Setting 2 (coefficient: 56,867; *p*-value: 0.05), but not in Setting 3 (coefficient: 3,620; *p*-value: 0.93). Subjects also reported their previous experience of betting on sports events or similar, as well as previous stock market experience. We combined this information to generate a binary variable proxying for experience of gambling and/or trading. We observe a positive relation between this self-reported measure of experience net profit in the experiment, but the effect is not statistically significant (coefficients in Setting 1 and 2, respectively: 19,225; *p*-value: 0.40 and 32,271; *p*-value: 0.29). Controlling for the aforementioned individual characteristics in the regression analysis does not affect our qualitative results.

## Discussion

Previous studies have examined several aspects of experimental asset markets, including the effect of private information [8], [9], [21]–[24], sequential information arrival [17], Bayesian updating [18], [25]–[29], and intellectual discovery processes [30]. We extend this research by studying prediction markets as they would be used in the practice of scientific research. We frame the information aggregation task as one of scientific discovery, include publication and use a computerized prediction market interface with an automated, subsidizing market maker.

We tested three settings that differed in the way that information was disseminated. In Setting 1, the information was always public, and mispricing was low. Because no trader ever had an information advantage relative to the other traders, profits arose from being faster than other traders at updating prices to incorporate new information, or from exploiting mispricing due to others' miscalculations. In Setting 2, different participants had different pieces of information that were private at all times. In this setting, mispricing in three of the six markets was comparable to the mispricing in the first setting. The other three markets, however, showed substantial mispricing at the end of the last trading round. Thus, information was not aggregated in an efficient, reliable manner in this setting. Nevertheless, participants could profit from an information advantage. In Setting 3, participants received private information that was subsequently made public. In this setting, mispricing was as low as in Setting 1. In contrast to Setting 1, but similar to Setting 2, there was a clear positive relationship between net profits and having an information advantage. Thus, the markets in Setting 3 combined the advantages of the two other Settings. Markets gave good forecasts about the probabilities of the hypotheses, while at the same time allowing participants to profit from information advantages. This indicates that combining publishing and prediction markets might be an attractive first step toward making prediction markets operational in science.

Publishing of information in the context of scientific research is a much more complex process than the one studied in our experiments. Therefore, further theoretical and empirical investigations will be required to study whether potential trading and publishing strategies are incentive-compatible under more realistic conditions. Our results can at best point to the potential benefits that might arise from combining publishing and trading. Despite their potential benefits, however, prediction markets on scientific issues are currently rare. Aside from regulatory problems that have been outlined recently [5], [31], this might be due to problems specific to the practice of science. In the following, we discuss problems that arise specifically for science applications.

Prediction markets function well when traders are numerous. Scientific expertise, however, is scarce. Consequently, liquidity constraints can be expected to be a problem for prediction market application in the context of scientific research. As our experiment shows, this problem can be mitigated through the use of automated market makers such as the logarithmic market scoring rule proposed by Hanson [15].

Participants' prior trading experience has been found to be an important determinant of market efficiency [9], [18], [21], [22], [26], [27]. In our study, familiarity with, or successful application of Bayes' rule showed a statistically significant positive correlation with the payoff of a participant. The correlation between profit and trading experience was positive but not statistically significant, probably because our experimental design was not as powerful for detecting experience-dependent effects as previous studies were. Researchers, however, cannot be expected to have extensive trading experience and experience with Bayesian inference, and may not be used to thinking about prices as information signals. This could be mitigated through some initial training. In addition, bets could be made by research groups, rather than individual researchers. Moreover, markets can be rational even if the participants, on average, are not: prices, and hence the market forecast, are driven by the marginal transaction and not by average beliefs [25], [32]–[34]. Our experiment indicates that when prediction markets are a complement to publication, markets might remain functional even if information is not revealed by a researcher to the market.

Mispricing may arise due to erroneous interpretation of market signals as well as strategic attempts of participants to mislead other traders. Uncertainty about the prevalence of traders with inside information may cause traders to incorrectly infer that an uninformed trade is actually an informed trade, and adjust their beliefs accordingly [35]–[37]. Recent theoretical work has shown that when information signals are not conditionally independent, prediction markets may not necessarily provide incentives for immediate and truthful revelation of information [20]. However, the potential impact of both misleading errors and misleading strategic behavior is diminished when information can be made public, suggesting that an added value arises from the combining prediction markets and conventional scientific publication. Participants may also attempt to manipulate the market in order to shed a favorable light on their own research. Experimental studies have shown that prediction market manipulation is difficult to achieve in practice [38]–[40].

Besides regulatory issues [5], [31], one of the major obstacle to a wide-spread use of prediction markets in science might come from difficulties to find suitable questions. Prediction markets perform best for contracts that are judged at a specific point of time in an objective fashion. Contracts on products from scientific research (“Will there be an FDA approved HIV vaccine by 2015?”) might therefore be suitable for trading on a prediction market and might disseminate valuable information to a broader public. Scientific hypothesis, on the other hand, can usually not be judged with absolute certainty based on a single event. Moreover, when tests are error-prone, absolute certainty is never attained. Scientific research usually develops a consensus about a theory or hypothesis, rather than absolute certainty, and this consensus typically emerges over time rather than due to a specific event.

One attractive option is to trade contracts on the outcome of an experiment. Contracts payoffs could, for example, depend on which of a set of competing hypotheses agrees best with the outcome of a specific experiment. This simply requires a suitable judgment technology that is agreed upon in advance and is common knowledge to the market participants. Alternatively, one could bet on the results that will be published by a specific point in time. This is particularly suitable for fields where experiments are registered before they are performed, and are standardized, so that overall estimates can be generated by pooling several experiments. The outcome of a set of clinical trials on the effect of a standard versus a novel treatment might, for example, be suitable for a prediction market. Based on our experiments one might speculate that such linking of markets and scientific publishing generates more reliable forecasts, as well as incentives for seeking an information advantage.

## Methods

### Participants

In total, 124 participants were recruited by the CLER-Lab at Harvard Business School. Most participants were students from the Boston area. Median age was 21, with approximately equal numbers of male and female participants. Participants received a performance-independent fee of $15 in addition to the payments earned in the experiment.

### Ethics Statement

The experiment was approved by Harvard University CUHS (F16041-101). Written informed consent was obtained from all participants.

### Hypotheses Used in the Experiments

The hypotheses are framed within the context of molecular biology. Suppose there are three genes (A, B, and C) that are known to interact in a linear biochemical pathway: The first gene activates the second, which in turn activates the third. The order of the sequence is unknown. Thus there are six possible pathways (ABC, ACB, BAC, BCA, CAB, and CBA) that form the set of hypotheses. Knowledge on these hypotheses can be characterized by six probabilities p(h_1_), … , p(h_6_).

### Information from Binary Tests

For identifying the correct hypotheses, participants receive pieces of information from binary tests. The results indicate whether or not a specific gene activates another, i.e. whether A activates B, A activates C, etc. Thus there are six different tests (AB, AC, BA, BC, CA, and CB). Each test supports two of the hypothesis, and each hypothesis is supported by two tests. A positive result on test AB, for example, supports the sequences ABC and CAB, while sequence ABC is supported by positive results on test AB and BC. All of the tests are equally prone to type I and type II errors, and the error rates were common knowledge to all participants. We used the error rates α  =  0.12 and β  =  0.3. These values are higher than the values of α<0.05 and β<0.2 that researchers traditionally aim to achieve in the life sciences. We use these error probabilities to ensure that in the experiments, participants are exposed to errors at a considerable frequency. After a test has been disclosed, the probabilities associated with the hypotheses can be updated according to Bayes' Theorem.

### Bayesian Updating

The posterior probabilities after test e_j_ are given by 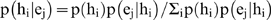 for a positive test result, and 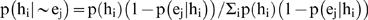 for a negative test result. The probability 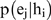 of getting a positive result on test j given that hypothesis i is true equals 1−β if test j supports hypothesis i, and α if it does not support hypothesis i.

### Trading Platform

Participants used a web-based prediction market to trade contracts representing the six hypotheses. After an initial instruction period on a practice market, each participant received login details for a trading account that was funded with 100,000 virtual money units (VMU). This endowment was equivalent to USD 10. Contracts for the correct hypotheses paid VMU 100 at the end of the experiment, contracts representing one of the false hypotheses paid VMU 0.

### Market Maker

The trading platform used an automated marker maker. This is an algorithm that offers a buying price and a selling price at all times, thus ensuring that there is always a counterparty with which to trade. The market maker takes a risk, because the net portfolio of claims it buys and sells typically do not cancel each other out. We used a logarithmic scoring rule as the basis for the market maker. The algorithm uses the net sales (S_1_, …, S_6_) the market maker has done so far for each of the six claims to determines the prices for a infinitesimally small trade in claim i as 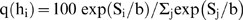. Parameter b determines liquidity and maximal subsidies provided by the market maker. We set the liquidity parameter to b = 2,000.

### Information Histories

Information histories were generated by randomly choosing one of the 6 tests for each round and subsequently generating the test result based on the error probabilities given above. The resulting six histories are: History 1: BA false, CB false, CA false, BC true, AC false, AB true, CB true; History 2: BC true, BC true, CB false, AC false, BC false, CB false, CB False; History 3: AB true, AC true, AB true, AB true. CB false, BA false, CA false; History 4: CB false, AB true, CA false, BC true, BA false, BA false, CB false; History 5: BA false, CB false, BA true, AB true, AB true, BC false, BC true; History 6: BA false, BC true, BA false, CB true, CB false, AB true, BC true. To avoid contamination between settings, we changed the labels on the tests and hypotheses between each market and setting.

### Informativity and Support in the Context of the Settings

Let **P** be the vector of probabilities (p(h_1_), … p(h_6_)) implied by the market prices. Suppose a trader whose beliefs are consistent with the current market **P** receives a new piece of information. He correctly updates his beliefs to **P′** and trades until market prices reflect his updated beliefs. If trading against a market maker like the one described in ref.[15] and used in our experiment, the resulting profit is 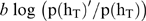, where h_T_ denotes the true hypothesis. Therefore, in Setting 2, one would expect a traders' profit to be proportional to the support 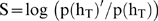, at least in the absence of budget constraints. Of course, the trader does not know the correct hypothesis, and therefore will expect a payoff of 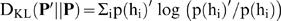. Note that while 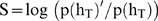 takes negative values for false findings, and positive findings for true findings, D_KL_ is always non-negative, even if a test result is erroneous. To realize the profit in Setting 2, a trader has to wait until the market is judged, because unwinding the new positions by selling contracts merely moves prices back from **P′** to **P**. In Setting 3, traders can in principle unwind their positions at a small loss once piece of information has been made public, because in contrast to Setting 2 other traders should keep the prices close to **P′**. Therefore, in this setting, traders could choose to realize a profit proportional to 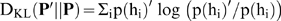 immediately after their information is disclosed, rather than waiting until the market is judged.

### Prior for Calculating Informativity and Support

Calculating S and D_KL_ requires a prior probability **P**. This prior could be taken from either correct Bayesian updating or from actual market prices. In a perfect market they are identical. In markets where mispricing is prevalent, it is suitable to use actual market prices because these prices likely provide a better representation of the actual beliefs of the traders. We therefore use market prices to calculate informativity I and support S of a test result.
